# Epileptic Seizure Prediction Based on Permutation Entropy

**DOI:** 10.3389/fncom.2018.00055

**Published:** 2018-07-19

**Authors:** Yanli Yang, Mengni Zhou, Yan Niu, Conggai Li, Rui Cao, Bin Wang, Pengfei Yan, Yao Ma, Jie Xiang

**Affiliations:** ^1^College of Information and Computer Science, Taiyuan University of Technology, Taiyuan, China; ^2^Centre for AI, Faculty of Engineering and IT, University of Technology Sydney, Sydney, NSW, Australia; ^3^Software College, Taiyuan University of Technology, Taiyuan, China

**Keywords:** epilepsy, electroencephalogram, permutation entropy, prediction, support vector machine (SVM)

## Abstract

Epilepsy is a chronic non-communicable disorder of the brain that affects individuals of all ages. It is caused by a sudden abnormal discharge of brain neurons leading to temporary dysfunction. In this regard, if seizures could be predicted a reasonable period of time before their occurrence, epilepsy patients could take precautions against them and improve their safety and quality of life. However, the potential that permutation entropy(PE) can be applied in human epilepsy prediction from intracranial electroencephalogram (iEEG) recordings remains unclear. Here, we described the novel application of PE to track the dynamical changes of human brain activity from iEEG recordings for the epileptic seizure prediction. The iEEG signals of 19 patients were obtained from the Epilepsy Centre at the University Hospital of Freiburg. After preprocessing, PE was extracted in a sliding time window, and a support vector machine (SVM) was employed to discriminate cerebral state. Then a two-step post-processing method was applied for the purpose of prediction. The results showed that we obtained an average sensitivity (SS) of 94% and false prediction rates (FPR) with 0.111 h^−1^. The best results with SS of 100% and FPR of 0 h^−1^ were achieved for some patients. The average prediction horizon was 61.93 min, leaving sufficient treatment time before a seizure. These results indicated that applying PE as a feature to extract information and SVM for classification could predict seizures, and the presented method shows great potential in clinical seizure prediction for human.

## Introduction

Epilepsy is an uncontrollable neurological disease that has a serious impact on patients, their families and society. It is characterized by sudden and recurrent seizures which are the result of an excessive and synchronous electrical discharge of a large number of neurons (Beghi et al., 2005). According to the estimations of the World Health Organization, around 50 million people worldwide suffer from epilepsy as the most common disorder of the brain activity (Gajic et al., 2015). Epilepsy can occur in all stages of life, drug and surgical treatments are often used to relieve the disease in the clinic (Xiang et al., 2015). However, approximately 30% of patients with epilepsy cannot be treated by either medication or surgery, and these patients must live with seizures that can occur anytime and anywhere (Fujiwara et al., 2016). Therefore, a successful system for predicting epileptic seizures is urgently needed for patients, and epilepsy prediction has become a very practical research topic (Leestma et al., 1984; Schelter et al., 2007).

In the 1970s, seizure prediction garnered much interest among scholars around the world, and significant research was performed from the perspective of dynamics and non-dynamics by analyzing electroencephalograms (EEGs) (D'Alessandro et al., 2003; Mirowski et al., 2009). Numerous experiments showed that the prediction of epileptic seizures is possible, as epileptic seizures develop over a long period of time and are not sudden. However, robust seizure prediction remains challenging, as the EEG patterns are not wide-sense stationary and change from seizure to seizure, from electrode to electrode, and from patient to patient (Perucca et al., 2014).

Seizure prediction uses pattern recognition methods to distinguish preictal samples from interictal samples in real time. Some studies have employed extensive methods to raise seizure alarms. Features are first extracted from the preprocessed, windowed EEG signals and then classified into preictal/non-preictal states. Finally, a seizure prediction algorithm is created to trigger a seizure alarm (Mirowski et al., 2009). In these methods, many feature types have been applied to enhance seizure predictability power, including the largest Lyapunov exponent (Iasemidis et al., 1990), correlation dimension (Lehnertz and Elger, 1998), dynamic similarity index (Le et al., 2003), entropy (Van et al., 2003; Li et al., 2007), and phase synchronization (Mormann et al., 2003; Kuhlmann et al., 2010).

The extracted features have an important influence on prediction performance. Permutation entropy (PE) has been used in several application to study epilepsy activity for determinism detection and dynamical changes in EEG signals. In the study with Mammone et al. (2015), PE was used to identify the different phases of epilepsy activity in childhood absence epilepsy. Bruzzo et al. applied PE to detect vigilance changes and preictal states from scalp EEG in epileptic patients. They evaluated the separability of the interictal and preictal phases was found, and PE was shown on be sensitive to changes in vigilance state (Bruzzo et al., 2008). Nicolau et al. investigated the use of PE as a feature for automated epileptic seizure detection (Nicolaou and Georgiou, 2012). Mateos et al. developed a method based on PE to characterize EEG from different stages in the treatment of a chronic epileptic patient (Mateos et al., 2014). These studies suggest that PE is a useful tool for the study of epilepsy.

PE was also used in prediction, Li et al. proved that PE can be used not only to track the dynamical changes of EEG data, but also to successfully detect pre-seizure states for the population of rats (Li et al., 2007). Compared with rats, human have great individual differences leading by various of ages, seizure focus, types of seizure and so on. Meanwhile, few studies have investigated PE as a tool to predict seizures from human by using intracranial electroencephalogram (iEEG) recordings. Therefore, it is necessary to explore the potential of PE in human. In the present study, we used an open-source database, provided by the Epilepsy Centre at the University Hospital of Freiburg, Germany. PE was used as the feature to analyze the transition process from normal to seizure state and a two-step post-processing method was used for epileptic seizure prediction.

The paper is organized as follows: the second section includes the data, specific method proposed and performance indices. The third section contains detailed experimental results. The fourth section analyzes the results. The last section discusses the conclusions of the study.

## Materials and methods

**Figure 1:**
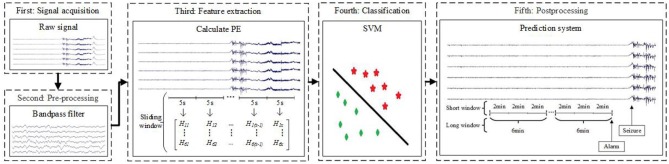
Block diagram of the proposed seizure prediction method. In the third block, *H*_*ab*_ (1 ≤ a ≤ 6,1 ≤ b ≤ *x*) represent PE. The letter a corresponds to the six channels, and the letter *x* is the number of samples after computing PE in a 5-s sliding window per channel.

Figure 1 presents a block diagram of the seizure prediction process. After preprocessing (the second block) from raw signals (the first block), we compute PE per channel considering an epoch (the third block). Then, we apply a SVM to learn a decision function based on training (the fourth block). After the learning phase, the testing set is classified using the trained model, and a post-processing stage is applied to generate alarms and reduces the influence of false positives (the fifth block). The following sections describe the methodologies applied in detail. In this paper, we use three different sliding windows: sliding window (5-s sliding window without overlap), short window (2-min sliding window without overlap) and long window (6-min sliding window without overlap).

### Dataset description and preprocessing

This paper uses the dataset recorded by the Epilepsy Centre at the University Hospital of Freiburg, Germany. The database contains iEEG recordings of 21 patients suffering from medically intractable focal epilepsy. The data were recorded during invasive pre-surgical epilepsy monitoring. To obtain a high signal-to-noise ratio and fewer artifacts and to record directly from focal areas, intracranial grid-, strip-, and depth-electrodes were utilized. The EEG data were acquired using a Neurofile NT digital video EEG system with 128 channels, 256 Hz (data from patient 12 was sampled at 512 Hz but downsampled to 256 Hz) sampling rate (Zhang and Parhi, 2016), and a 16-bit analog-to-digital converter. Patients with fewer than three seizures were not analyzed in this paper because training using preictal data from only one seizure is likely to lead to model overfitting to that particular seizure and may not be able to predict others. Therefore, at least two seizures must be selected in the training set and the other seizure is used for testing (Zhang and Parhi, 2016). All patients in the experiment had 3–5 seizures. The dataset contains 83 seizures from 19 patients, except for patients 8 and 13, who did not qualify. The subjects are in the 10–50 age range with eleven women and eight men. There are three different seizure types, including simple partial(SP), complex partial(CP), generalized tonic-clonicand(GTC) and everyone has at least two types. Among these patients, the epileptic focus of 10 patients was in neocortical brain structures, seven patients in the hippocampus, and two patients in both. The seizure onset times and epileptiform activities were annotated by certified epileptologists from the Epilepsy Centre. The 50 Hz power-line interference is removed from the raw EEG data by applying a notch filter.

### Feature extraction and proposed system

#### Feature extraction

After preprocessing of the EEG segments, PE was computed for each EEG channel to measure the complex degree of a time series (Bandt and Pompe, 2002). The feature is computationally efficient, with potential for real-time implementation. There are other three types of PE measures involved in this study, including Tsallis PE(TPE), Renyi PE(RPE) (Liang et al., 2015) and Permutation min-entropy(MPE) (Zunino et al., 2015). Here, we present PE based on Shannon entropy, results for other three measures can be seen in Supplementary Material. The specific calculation process of PE is as follows:

For a given scalar time series {*x*(*i*), 1 ≤ *i* ≤ *N*}, a vector composed of the m-th subsequent values is constructed as follows:

(1) $$\begin{array}{l} \;X(1)=\{x(1),x(1+\lambda ),\cdots ,x(1+(m-1)\lambda )\}\\ \;\vdots \\ \;X(i)=\{x(i),x(i+\lambda ),\cdots ,x(i+(m-1)\lambda )\}\\ \;\vdots \\ X(N-(m-1)\lambda )=\{x(N-(m-1)\lambda ),x(N-(m-2)\lambda ),\cdots ,x(N)\} \end{array}\}$$

where *N* is the length of a given scalar time series, *m* is the embedding dimension, λ is the delay time, and *X(i)* is the i-th component.

Reordering *X(i)* to an ascending order:

(2) $$X(i)=\{x(i+(j_{1}-1)\lambda )\leq x(i+(j_{2}-1)\lambda )\leq \cdots \leq x(i+(j_{m}-1)\lambda )\}$$

where *j* = 1, 2, ⋯, *m*. When two elements are equal, we order according to the values of their corresponding *j*.

We obtain a set of symbol sequences by each row of the reconstructed matrix of any time series, with sequences such as *S*(*l*) = {*j*_1_, *j*_2_, …*j*_*m*_}, (*l* = 1, 2, …*k, k* ≤ *m*!) where *k* is the objective quantity of {*j*_1_, *j*_2_, ⋯*j*_*m*_}. In the sequel, any vector *X(i)* is uniquely mapped to (1, 2, …, *m*) or (2, 1, 3, …, *m*)… or (*m, m*−1, …, 2, 1) in a total of *m!* different situations.

Let the probability of occurrence of distinct symbols be:

(3) $$P_{g}(g=1,2,\cdots k)$$

The PE of order *m* ≥ *2* is defined as the Shannon entropy for the *k* distinct symbols:

(4) $$H_{p}(m)=-\sum_{g\;=1}^{k}P_{g}\ln P_{g}$$

(5) $$H_{p}=H_{p}(m)/\ln(m!)$$

PE measures the departure of the time series from a completely random process: a smaller PE indicates a more regular time series. The embedding dimension *m* = 3, 4…, 7 have been recommended. In this experiment, we selected *m* = 4 and λ = 1 to extract most information. The selection of the appropriate parameters is based on related experiments. The details of the selection process is shown in Supplementary Material. In addition, the parameter pairs is also chosen in a similar study (Li et al., 2007).

In this study, PE was computed using a 5-s sliding window without overlap (Cook et al., 2013; Bandarabadi et al., 2015). For the purpose of seizure prediction, we consider a 5-s interval suitable to represent variations in the iEEG data that is a good compromise between a longer window and the need to assume stationarity of the EEG segment (Direito et al., 2017).

If the length of raw time series is *L* for each channel, the number of samples after computing PE is:

(6) $$x=\left\lfloor \frac{L}{5*256}\right\rfloor$$

#### Classifier training

Following the feature extraction stage, each 5-s EEG segment described by a set of features was labeled as one of two possible states: preictal or interictal. For classification, a classifier should be able to improve prediction performance because it takes all extracted features into consideration simultaneously. SVM has been extensively used in the current study (Aaruni et al., 2015; Song and Zhang, 2016). And it is considered the most powerful and favorable classifier in binary classification (Vapnik, 2002; Park et al., 2011).

The dataset of each subject was divided into two parts: a training set and a testing set. The testing set was not used in the optimization of the SVM models. In terms of the number of samples per class, the training set is highly unbalanced. To alleviate the effect, we undersample the inter-preictal set by random selection (Chawla et al., 2004). In this study, we use the RBF as the kernel. The RBF kernel is used since it can nonlinearly map the data into a higher dimensional feature space. The linear kernel is a special case of RBF (Fu et al., 2015). In the study by Babak Sharif, who also used the Freiburg EEG dataset, a detailed explanation is given for the use of RBF (Sharif and Jafari, 2017).

The SVM complexity parameter *C* is known as cost and represents the tradeoff between the classification margin and non-separable patterns. To prevent the overtraining of classifiers, it is estimated via six-fold cross validation of the training set. The parameter *g* defines the influence of a single training example; low values mean “far” and high values mean “close.” The *(C,g)* is optimized using a grid search in our experiment (Liu et al., 2010; Kaya and Kaya, 2014).

#### Postprocessing

**Figure 2:**
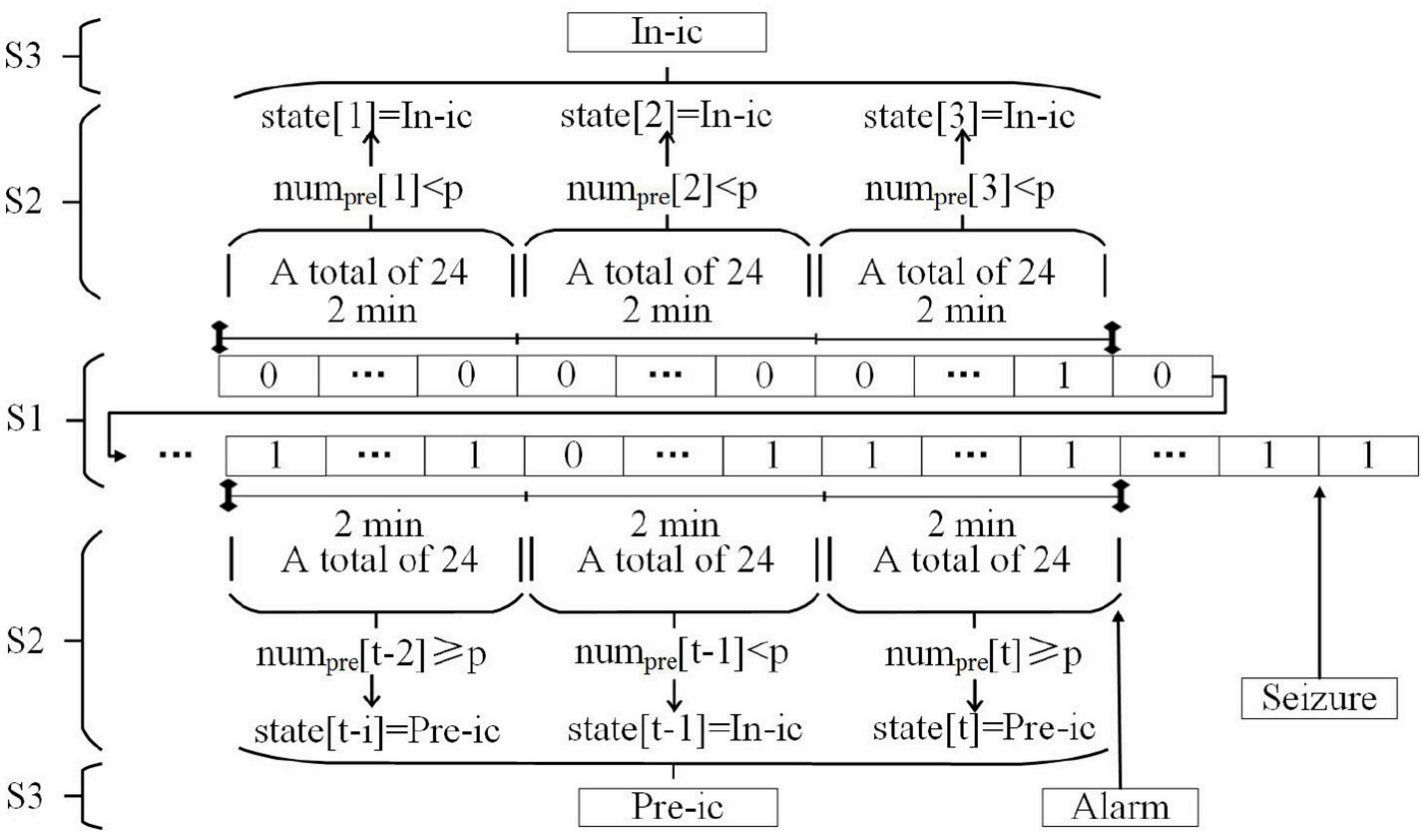
Prediction system. In-ic = Interictal; Pre-ic = Preictal.

After training the learning model, a prediction system is used to transform the output of the model into an appropriate alarm generator. In theory, an alarm could be raised whenever the output of the model is preictal. However, this behavior is unrealistic in actual scenarios. A firing power (FP) approach is more “conservative” for raising alarms due to its particular constraints on the times when alarms are possible (Teixeira et al., 2012). The post-processing method we use is similar to the FP approach. Here, we provide Figure 2 to illustrate the prediction process. The meanings of expressions and letters in the figure is as follows. First, the output of the SVM classifier is transformed into a binary label that binaries the output according to:

(7) $$tg_{i}=\{\begin{array}{ll} 1, & \text{if preictal}\\ \text{0}, & \text{if interictal} \end{array}(1\leq i\leq x)$$

where *tg*_*i*_ is the label of the *i*-th 5-s sliding time window, and *x* corresponds to formula (6). For S1 in Figure 2, there are *x* rectangles, and each rectangle represents 5 s in the raw signals. The numbers 0 and 1 in each rectangle correspond to *tg*_*i*_.

Second, a 2-min short window was set to quantify the number of samples classified as preictal according to:

(8) $$num_{pre}[t]=\sum_{m(t-1)+1}^{mt}tg_{i}$$

*m* is 24 because 2 min contains 24 sliding windows when computing the feature, and *num*_*pre*_*[t]* corresponds to the number of preictal samples at the t-th short window.

The states can be determined in the 2-min window using the *num*_*pre*_*[t]* according to:

(9) $$state[t]=\{\begin{array}{ll} Preictal, & if\;num_{pre}[t]\geq p\\ Interictal, & if\;num_{pre}[t]<p \end{array}$$

where *p* is an arbitrary threshold value in reality, which in this work assumed the values {1, 2, …, 11, 12}, and *state[t]* represents the condition of the t-th short window. In other words, if *num*_*pre*_[*t*] ≥ *p*, the subject stayed in the preictal period for the past 2 min. This step corresponds to S2 in Figure 2.

Finally, a long window of 6 min (formed by combining three consecutive short windows) is applied for a final decision to obtain a lower false prediction rate. If at least one (or two or three, depending on the subject) of the three consecutive short windows is classified as preictal, the system makes actual predictions and an alarm is generated. For S3 in this figure, we assume that when at least two short windows are preictal, an alarm is generated.

In post-processing, a short window (2 min) and a long window (6 min) are used to predict an impending seizure by analyzing preictal and interictal EEG signals. This process is called a two-step FP method in the rest of this paper. Only a one-step time window is applied in the original FP method, i.e., the *state[t]*, to make a final decision for the following segments. We contrasted both two-step FP (short window = 2 min, long window = 6 min) and one-step FP (the unique window = 6 min) in our study.

**Figure 3:**
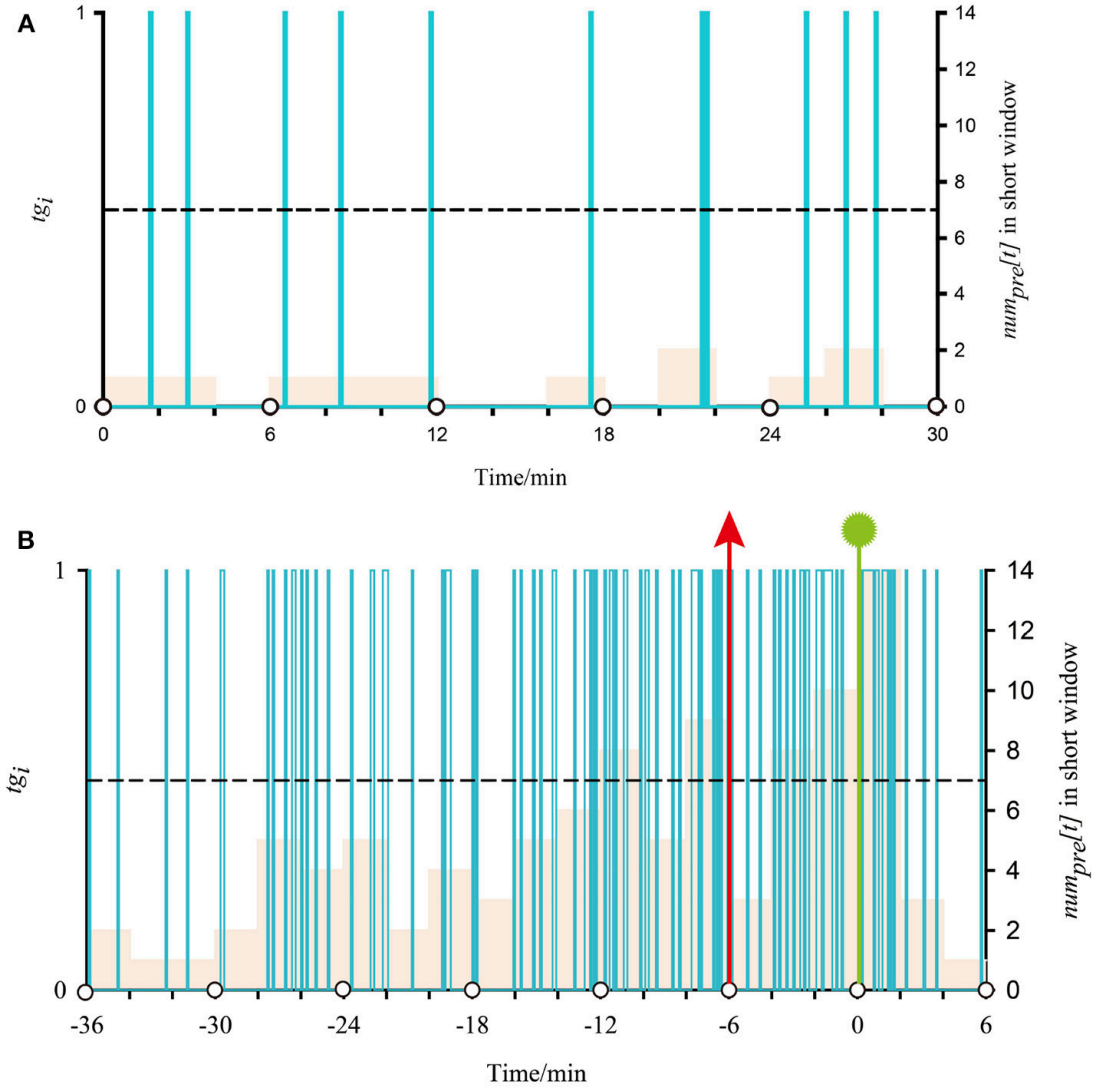
Demonstration of classified output after SVM classification and proposed regularization using iEEG signals from the first seizure of patient 17. **(A)** Shows a normal situation with no alarm in two-step FP. **(B)** Shows the application of post-processing during the preictal period in two-step FP.

Here, we use the first seizure of patient 17 as an example to illustrate the application of two-step FP and one-step FP on SVM outputs. In Figure 3, the time interval between two white circles on the horizontal axis represents a long window, and the interval of two adjacent scales represents a short window. Purple columns represent the classification output using SVM [*tg*_*i*_ in formula (7)]. Its value corresponds to the main vertical axis. The almond columns represent the number of samples classified as preictal in the short window (*num*_*pre*_*[t]* in formula (8)), and its value corresponds to the minor vertical axis. The black dotted lines represent the threshold lines, i.e., *p* in formula (9) (threshold = 7, the best model). When at least two almond columns in a long window appear across the threshold lines, there will be an alarm. Seizure onset is marked by the vertical green line with a polygonal star. The vertical red arrow represents an alarm. In Figure 3B, the 0-point on the horizontal axis indicates the beginning of the long window in which the seizure begins. The negative value on the horizontal axis indicates the preictal segments.

Figure 3A, time 30 min, presents a normal situation that does not generate an alarm. In all almond columns, the maximum value is 2, below the threshold line; i.e., no short window is considered preictal. As there is no preictal state during this time, there is no alarm. For Figure 3B, there are four times that the almond columns cross the threshold line before onset, and the first two are in a long window. According to the best model, if at least two short windows are considered preictal in a long window, an alarm will be generated. In other words, there is an alarm at (−6) min (vertical red arrows) during the preictal period, and seizure onset at 0.548 min (vertical green line with polygonal star). The alarm outside the preictal period is considered a false alarm.

### Prediction performance indices

To evaluate the method, we measured seizure prediction horizon (SPH), sensitivity (SS), and false prediction rates (FPR). SPH is defined as the time interval between the predictive alarm and seizure onset. SS is the ability of the system to correctly predict seizures and is defined as:

(10) $$SS=N_{c}/N_{t}$$

where *N*_*c*_ is the number of correctly predicted seizures, *N*_*t*_ is the total number of seizures.

FPR corresponds to the number of false predictions per time interval. It can be defined as:

(11) $$FPR=N_{f}/NT$$

Where *N*_*f*_ is the number of inaccurately predicted seizures, and *NT* is the total time of EEG signals.

SS and FPR are the popular criteria used to evaluate performance of the techniques for prediction of epileptic seizure (Parvez and Paul, 2016).

In order to find the best trained SVM model for each patient, the Euclidean distance is calculated according to another study (Bandarabadi et al., 2015). The best model is selected by the minimum Euclidean distance. The specific calculation process of distance is as follows:

1. To limit the effect of narrow range and very low FPR(FPR is easily near 0), the normalized FPR is used instead of the actual FPR. The normalized FPR is defined as:
  (12) $$fpr_{n}(u)=\frac{fpr(u)}{fpr_{\max}}*100,\;u=1,\ldots ,n$$
  (13) $$fpr_{\max}=\max(fpr(u)),\;u=1,\ldots ,n$$
  where the *fpr(u)* is the FPR of the *u*-th SVM model, and *n* is the number of SVM used.
2. The Euclidean distance is calculated as:
  (14) $$ed(u)=\sqrt{{\left(ss(u)-100\right)}^{2}+{\left(fpr_{norm}(u)\right)}^{2}},\;u=1,\ldots ,n$$
  where the ss(u) is the SS of the *u*-th SVM model. We select the SVM model which provides the minimum *ed(u)*.

## Results

We tested our patient-specific seizure prediction method on the iEEG of interictal recordings and 19 patients with 83 seizure events. Here, we use some datasets as examples to present the results.

### Results of feature extraction

**Figure 4:**
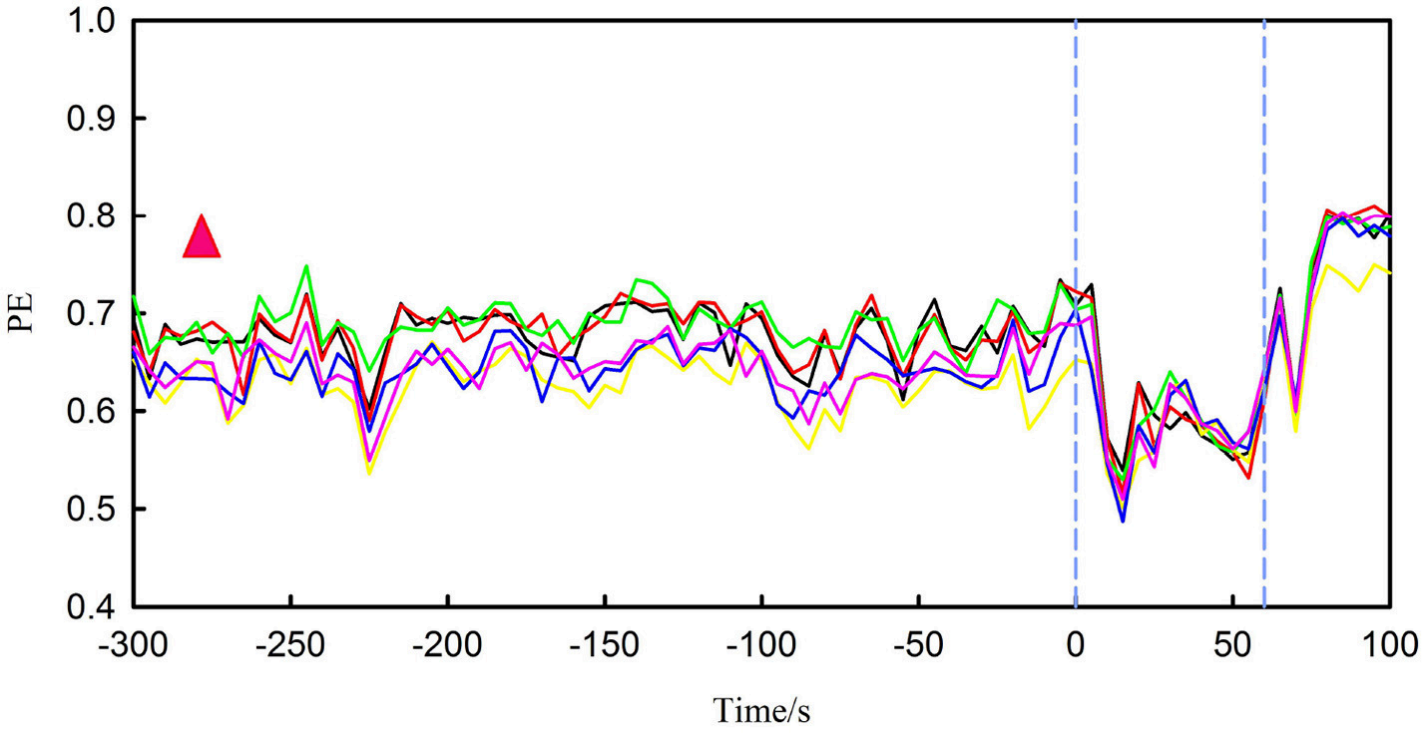
It represents PE for the first seizure of patient 17, in which the interval between the two blue vertical lines is ictal period. There are six different lines, each colored broken line represents changes in PE for one channel. The red triangle represents the range of PE during the interictal period.

Figure 4 shows PE for the first seizure of patient 17 (this dataset has five recorded leading seizures). The length of time presented in the figure is extracted from the 300 s before onset until the end of the seizure. From the figure, the PE of the preictal segment (before the first blue lines) is between approximately 0.5 and 0.75. In terms of the patient, the feature rises at first and then sharply declines in the ictal period (interval between two blue vertical lines). The other four seizures also have similar changes. The PE during interictal period is between 0.75 and 0.8, higher than the preictal period and ictal period. So the behavior of this feature changed dramatically from interictal period to ictal period. We also observed noticeable changes for other patients.

### SS and FPR of all patients

Table 1 reports the best results for each patient in one-step FP and two-step FP, obtained from the proposed methods. The best results were selected so that SS and FPR can be near-optimal (SS = 100%, FPR = 0 h^−1^). Using the two-step FP method, an average of 94.0% of seizures were successfully predicted, with an average FPR of 0.111 h^−1^. The best predictions with SS of 100% and FPR of 0 h^−1^ were achieved, such as in patient 4, to reach an ideal state. Of course, some patients had poor results, such as patient 5. Both SS and FPR have very unsatisfactory results with 80.0% and 0.525 h^−1^. Overall, the one-step FP method can predict 93.8% of seizures correctly and 0.423 h^−1^ FPR was obtained.

**Table 1:** Results for all patients in two-step FP and one-step FP.

**Patient ID**	**Two-step FP**				**One-step FP**			
	**SS**	**FPR**	**Average SPH (min)**	**Distance**	**SS**	**FPR**	**Average SPH (min)**	**Distance**
1	1.000	0.281	59.00	15.25	0.906	0.406	58.97	18.25
2	0.708	0.042	49.90	29.28	0.708	0.708	56.60	39.45
3	1.000	0.125	63.02	9.26	0.950	0.275	64.52	14.96
4	1.000	0.000	60.17	0.00	1.000	0.000	60.17	0.00
5	0.800	0.525	55.75	36.42	0.825	1.000	58.27	42.94
6	1.000	0.167	59.76	6.67	1.000	1.292	63.01	36.90
7	1.000	0.042	63.26	4.17	1.000	0.458	71.26	25.58
9	1.000	0.000	67.21	0.00	1.000	0.775	67.21	51.67
10	1.000	0.000	75.57	0.00	1.000	0.125	77.52	7.25
11	0.781	0.063	54.23	22.04	0.781	0.250	59.75	23.58
12	1.000	0.125	34.50	26.67	1.000	0.125	34.50	26.67
14	1.000	0.063	48.87	4.65	1.000	0.406	62.74	28.89
15	1.000	0.000	59.38	0.00	1.000	0.438	67.82	15.56
16	0.800	0.200	58.63	24.89	0.800	0.325	64.07	28.28
17	1.000	0.000	77.66	0.00	1.000	0.275	85.46	20.37
18	0.925	0.075	64.29	10.89	0.950	0.250	67.16	10.23
19	0.875	0.250	65.55	17.85	0.906	0.375	62.17	18.44
20	0.975	0.150	79.35	9.70	1.000	0.450	79.54	20.00
21	1.000	0.000	80.53	0.00	1.000	0.100	81.88	5.88
Avg.	0.940	0.111	61.93	11.49	0.938	0.423	65.40	22.86

### SPH of all patients

**Figure 5:**
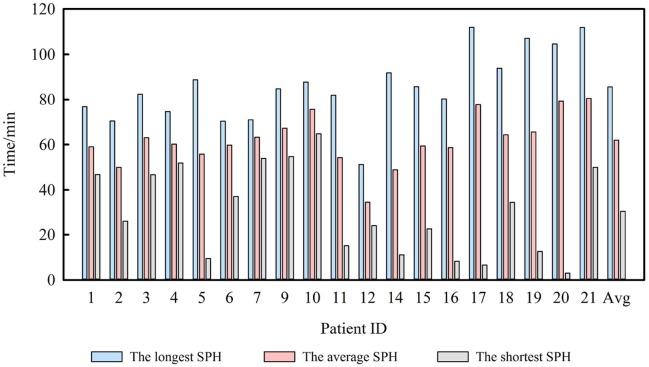
SPH and average SPH for 19 patients.

Table 1 also shows the average SPH for each patient. In two-step FP, the longest average SPH was 80.53 min, and the shortest was 34.50 min. For all seizures, the average SPH was 61.93 min. In addition, almost all patients had an average predicting time of 40 to 80 min according to Figure 5. Thus, our method successfully distinguished iEEG at 40–80 min prior to seizures from interictal recordings. In one-step FP, the average SPH was 65.40 min.

### Euclidean distance

**Figure 6:**
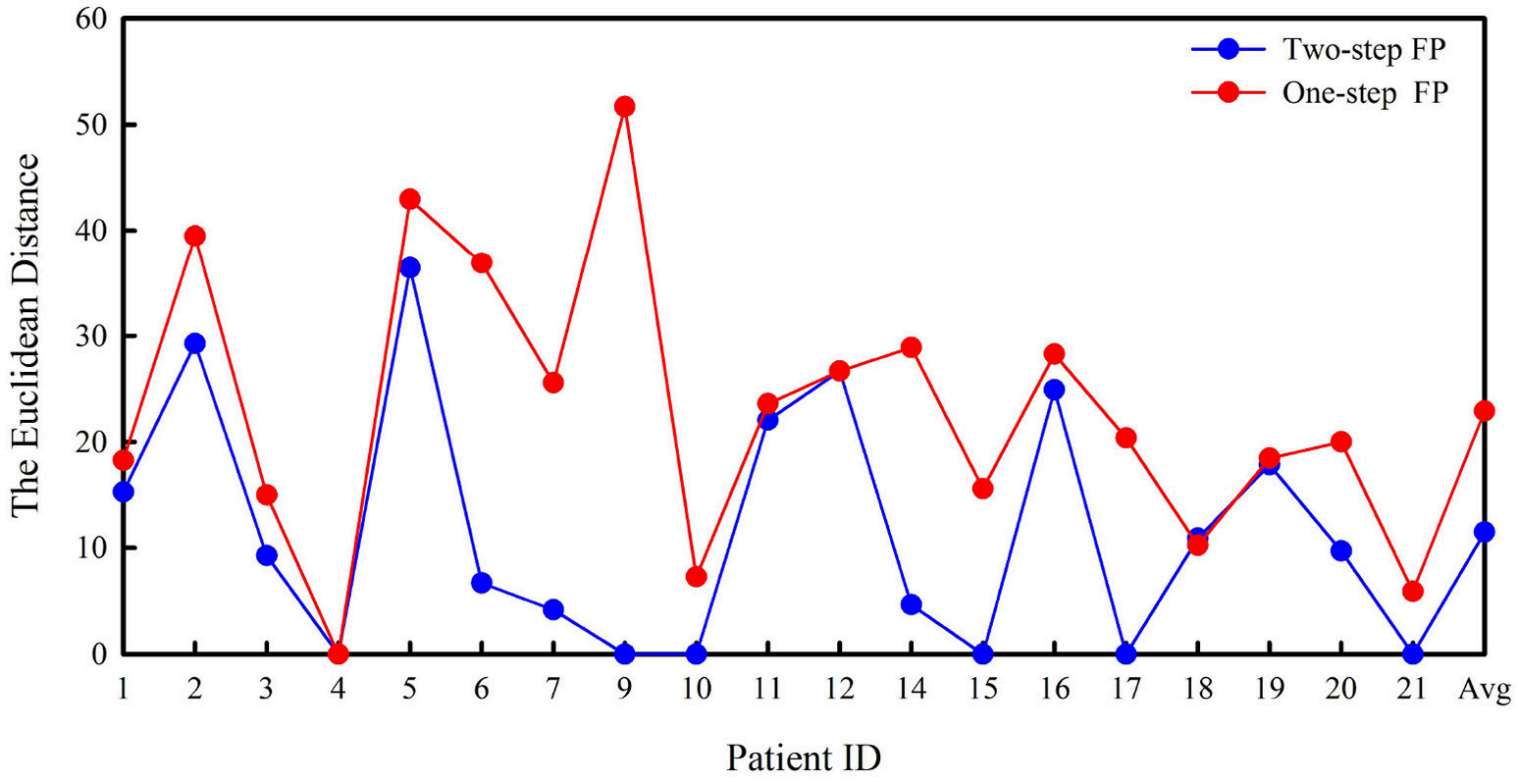
Euclidean distance in two-step FP and one-step FP.

Figure 6 shows both the minimum Euclidean distance for each patient selected by the best model and the average distance. The average distances are 11.46 in two-step FP and 22.89 in one-step FP.

## Discussion

### The advantages of PE

Feature extraction covers linear and nonlinear methods recently (Navarro et al., 2002). The linear method usually has a lower computational burden, but it has poor robustness which makes it difficult to show a great advantage in reflecting the dynamic changes of the brain electrical signals. Compared with linear analysis methods, nonlinear analysis methods show higher classification accuracy and better robustness in previous studies. The entropy of the EEG may act as a reliable indicator of changes in cortical neuronal interactions and truly reflect the intra-cortical information flow. PE has some advantages over other commonly used entropy measures, such as approximate entropy and sample entropy, including its simplicity, low complexity in computation without further model assumptions, and robustness in the presence of observational and dynamical noise (Bandt and Pompe, 2002). It is also a feature with only one dimension, So the proposed method may be highly appropriate for online and real-time analysis.

In previous studies, some researchers have focused on the combination of various features to improve prediction performance. However, multiple features may lead to lower time efficiency. For example, Zheng's study showed that the combination of phase synchronization and spectral power features is unnecessary due to increased computation complexity (Zheng et al., 2016). That is, multiple features may not always be necessary. In this work, the PE is applied as the unique feature. So the fewer features can also decrease the computation cost.

### Subject-specific modeling

An important property of seizure prediction is subject-specific data-analytic modeling. Hence, we expect that prediction quality varies with subject due to the varying quality of preictal data for different subjects (Shiao et al., 2016). In this work, all interictal and preictal recordings for particular subject were divided into training and testing sets, and the classifier was trained with these particular data. No cross-testing was done for the data from other subjects. After training, the parameters giving highest performance were obtained for post-processing. In other words, the p (in formula 9) varied from patient to patient in the short window of two-step FP. If one/two/three consecutive short windows are referred to as preictal, an alarm is generated. These practices consider differences between patients for better clinical considerations.

### Two-step FP vs. one-step FP

In the exploration stage of the study, we also set a 2-min window in one-step FP, and the results revealed many isolated mispredictions despite the relatively high SS. These results showed that 2 min is not sufficient to accurately determine the current state for a patient. When a 6-min sliding window is applied, although the performance improved significantly, the FPR was still unsatisfactory. Therefore, two-step FP was used in the study.

According to our results, two-step FP gave better predictions for almost all test segments or patients than one-step FP. In addition, the three indices showed better performance for all patients, except for SPH. In particular, the average FPR in our results is satisfactory and lower than the chance level of 0.15 h^−1^ (Sharif and Jafari, 2017). Some patients have the same accuracy using both methods, but the two-step FP method has a lower false alarm rate than the one-step FP method, e.g., in patient 7. Almost all patients have higher SS or lower FPR after applying two-step FP than one-step FP. According to Figure 6, two-step FP is always equal or lower than one-step FP except for patient 18 in terms of Euclidean distance. For average SPH, two-step FP is slightly shorter than one-step FP, but it performs well enough for clinical application. The results for SPH coincide with previous studies, and the interval can leave enough time for clinical treatment (Chisci et al., 2010; Williamson et al., 2012).

### Comparison with others

Many other methods are also proposed by other researchers for epileptic seizure prediction. Table 2 compares the results of the proposed method and other methods. Only methods using the same data are incorporated for a realistic comparison.

**Table 2:** Comparison to prior work.

**References**	**SS/FPR**
Sharif and Jafari, 2017	91.8–96.6/0.05-0.08
Zheng et al., 2014	80/0.17
Williamson et al., 2012	90.8/0.095
Aarabi and He, 2014	92.6/0.15
Ayinala and Parhi, 2012	94.37/0.14
Ozdemir and Yildirim, 2014	96.55/0.21
Park et al., 2011	97.5/0.27
Wang and Lyu, 2015	98.8/0.054
Chisci et al., 2010	100/0.17
This work	94/0.111

According to Table 2, compared with studies of Zheng et al. and Aarabi et al. our study obtained better SS and FPR. Although some studies obtained better SS (Chisci et al., 2010; Park et al., 2011; Ayinala and Parhi, 2012; Ozdemir and Yildirim, 2014), we obtained a lower FPR. Similarly, Williamson's study (Williamson et al., 2012) had lower FPR, but we got higher SS. Wang's prediction systems and Sharif's method involved a much larger number of features than this work, and finally, achieved better SS and FPR. For a single feature, it has some limitations in extracting features. This work has realized equal results with other studies to some extent. In comparison, the method proposed in this study not only demonstrated equal overall performance, but also produced feature vectors with lower dimension.

Existing literature illustrated that the time range of prediction is from few seconds to tens of minutes. In the study with Li et al., the average anticipation time is around 4.9 s for 28 rats with absence seizures (Li et al., 2007). Navarro et al. obtained a mean anticipation time of 7.5 min in human neocortical partial epilepsy (Navarro et al., 2002). Van Drongelen et al. even got longer anticipation time and the maximum time is 40 min (Van et al., 2003). All these studies were based on a specific type of seizure and the results were significantly different. So different type of seizure may have different challenge in prediction time. Some other factors (like ages, sex, seizure focus) may also lead to different forecast time. In this study, although every patient has great individual differences, we still realized longer forecast time than other methods based on the same data.

## Conclusions

This paper investigated the predictability of an epileptic seizure from iEEG for human. A prediction model based on PE and nonlinear SVM was obtained for each patient. To accurately evaluate the predictive analytics, we explored the methods by considering specific patients. The predictability varied significantly across patients, demonstrating the variety of abnormal brain activities and potential advantages of patient-specific methods for seizure predictions.

The present study was limited by some factors. One persistent difficulty in assessing seizure prediction algorithms is the scarcity of long-duration recordings with an adequate number of spontaneous seizures and adequate duration of interictal data. In addition, the non-abruptness phenomena and inconsistency of the signals, along with different brain location, patient age, patient sex, and seizure type, are challenging issues that affect the consistency of performance in terms of advanced SS and FPR with existing methods for all types of patients. Further study is necessary to determine the cause of low predictability for some patients. We are currently exploring the method that can potentially apply in real-time seizure prediction.

## Author contributions

YY and MZ are co-first authors and completed the entire study of the experiment and writing. YN, CL, RC, and BW revised the manuscript. PY and YM provided advice and guidance. JX provided the research ideas.

### Conflict of interest statement

The authors declare that the research was conducted in the absence of any commercial or financial relationships that could be construed as a potential conflict of interest.
