# Supplementary material for: Epileptic Seizure Prediction Based on Permutation Entropy
Source: Front Comput Neurosci. 2018 Jul 19;12:55. doi: 10.3389/fncom.2018.00055 (PMC6060283; doi:10.3389/fncom.2018.00055)
Supplement: Supplementary file 1 [file Data_Sheet_1.DOC]

**Supplementary Material**

1. Parameters Selection

Permutation entropy(PE) contains several parameters, which may severely impact the output of experiment. Therefore, it is very important to select the appropriate parameters. There are other three types of PE measures involved in this study, including Tsallis PE(TPE), Renyi PE(RPE) and Permutation min-entropy(MPE). In order to distinguish from other PE, we call Shannon PE(SPE) for permutation entropy([Bandt and Pompe, 2002](#_ENREF_5)) . There are two parameters(embedded dimension $m$, delay time λ) which need to be considered for SPE and MPE. For TPE and RPE, we need to discuss three parameters(embedded dimension $m$, delay time λ , q).

In this study, the method of select the parameters is based on classification performance, including sensitivity, specificity and accuracy. A six-fold cross validation was selected to examine the classification performance, i.e., the sample set was divided into a set of six disjointed subsets with the number of samples in each subset roughly equivalent. The five training subsets were selected for training the classification model, in which the optimal parameters of the kernel function in the SVM model were identified using a grid searching optimization method. Subsequently the left subset was used for verifying the performance of the developed classification model. The above process was repeated ten times, and thus, each subset was involved in the detection.

These three indexes, accuracy, sensitivity and specificity, can be calculated as follows:

|  | $Sensitivity=\frac{TP}{TP+FN}$ | (1) |
| --- | --- | --- |
|  | $Specificity=\frac{TN}{FP+TN}$ | (2) |
|  | $Accuracy=\frac{TP+TN}{P+N}$ | (3) |

P denotes the number of samples during a preictal period; N denotes the number of samples during a interictal period; FP denotes the number of samples for a interictal period but are mistaken for a preictal period; FN denotes the number of samples at a preictal period that are mistaken for a interictal period; and TP and TN denote the numbers of samples which are accurately classified.

1. Discuss λ

Table 1 shows the average classification performance of four entropy measures(SPE, TPE, RPE, MPE) by using different parameter pairs(m, λ). It can be seen that the SPE achieved best results(It is marked in blue font) according to accuracy when m equals 4 and λ equals 1. Similarly, TPE, RPE and MPE can achieve best results when m equals 3 and 7, λ equals 1. It is hard to see a significant difference in the overall results. However, four types of PE all worked better when λ is equal to 1(The best results were marked in blue font). So λ=1 was selected in subsequent experiments.

1. Discuss m

Table 2 shows the best m of four measures when patients obtained their own best classification results by using λ=1. Based on this table, we can calculate separately how many patients got the best results for each parameter pairs and the results can be seen in Table 3. For example, there are seven patients who achieved best results when m=3 and λ=1. As we can seen, more patients obtained best results when m=4 and λ=1 for SPE. Here, we recommend m=3 and m=4, because they got equal results to a certain degree for SPE. When m=3 and λ=1, there are more patients who got best results for TPE and we also recommend m=7. With m=7 and λ=1, more than half of patients performed better for RPE and MPE. Therefore, m=4 and λ=1 was selected for SPE. Similarly, m=3 and λ=1 was more appropriate for TPE. For RPE and MPE, m=7 and λ=1.

1. Discuss q

For TPE and RPE, another parameter q(0<q<1, q>1) also needs to be discussed. Here, we concerned 0<q<1and 1<q<10. Table 4 shows all results for TPE (m=3,λ=1) and RPE(m=7,λ=1). It is suggested that q=2 is best for them and this parameter was applyed in subsequent experiments.

1. The parameter pairs selected in our study

Based on the above analysis, we used the following parameters for the experiment: SPE(m=4,λ=1;m=3,λ=1); TPE(m=3,λ=1,q=2); RPE(m=7,λ=1,q=2); MPE(m=7,λ=1).

1. The prediction results of four measures

Table 5 shows the prediction results for all patients by using two different parameter pairs(m=4,λ=1 and m=3,λ=1) for SPE. Table 6 shows the prediction results for all patients by using most suitable parameter pairs for TPE(m=3,λ=1,q=2), RPE(m=7,λ=1,q=2) and MPE(m=7,λ=1). From these results, sensitivity (SS) and average seizure prediction horizon (SPH) did not have significant changes. However, false prediction rates (FPR) and Euclidean distance were affected to some extent. By contrast, SPE achieved the best results when m= 4 and λ=1. The reason for different parameter pairs lead to different FPR is that patients with epilepsy always have very large individual differences caused by age, type of seizure, seizure focus and so on. Different patients may be suitable for different parameter pairs. It is undeniable that some patients still achieved good results such as patient 4, 18 when m= 3 and λ=1 for SPE. For other three types of PE, we can also find similar results.

In summary, different parameter pairs and algorithms indeed have a certain influence on the prediction results. However, there are several patients who can achieve good results in several situations, so it is difficult to say that which measure or parameter pairs is the best choice for all patients. Taking into account the operability of applications in reality, it may be better to use SPE and MPE as they have fewer parameters. Compared with MPE, SPE has achieved better results.

Table 1 The average classification results for all patients by applying different m and λ

| **Parameters** | | **SPE** | | |  | **TPE** | | |  | **RPE** | | |  | **MPE** | | |
| --- | --- | --- | --- | --- | --- | --- | --- | --- | --- | --- | --- | --- | --- | --- | --- | --- |
| m | λ | Sensitivity | Specificity | Accuracy |  | Sensitivity | Specificity | Accuracy |  | Sensitivity | Specificity | Accuracy |  | Sensitivity | Specificity | Accuracy |
| 3 | 1 | 89.30 | 80.75 | 84.99 |  | **89.30** | **81.87** | **85.55** |  | 88.78 | 80.81 | 84.74 |  | 84.01 | 75.51 | 79.72 |
| 3 | 2 | 89.11 | 80.49 | 84.75 |  | 89.12 | 80.44 | 84.73 |  | 88.94 | 80.74 | 84.82 |  | 86.43 | 77.41 | 81.86 |
| 3 | 3 | 87.72 | 80.10 | 83.85 |  | 87.77 | 80.08 | 83.86 |  | 87.71 | 80.20 | 83.91 |  | 85.53 | 77.52 | 81.46 |
| 3 | 4 | 85.37 | 79.37 | 82.33 |  | 85.35 | 79.32 | 82.29 |  | 86.16 | 79.53 | 82.72 |  | 84.15 | 76.56 | 80.31 |
| 3 | 5 | 84.65 | 78.41 | 81.51 |  | 84.56 | 78.47 | 81.49 |  | 85.23 | 78.96 | 82.05 |  | 82.26 | 76.76 | 79.46 |
| 4 | 1 | **89.25** | **80.81** | **85.01** |  | 88.50 | 80.31 | 84.38 |  | 89.00 | 80.53 | 84.71 |  | 86.84 | 78.33 | 82.52 |
| 4 | 2 | 88.85 | 80.47 | 84.64 |  | 88.89 | 80.07 | 84.45 |  | 88.98 | 80.89 | 84.89 |  | 87.62 | 79.06 | 83.27 |
| 4 | 3 | 87.74 | 80.38 | 83.99 |  | 87.58 | 80.34 | 83.88 |  | 87.01 | 81.25 | 84.04 |  | 85.49 | 79.56 | 82.51 |
| 4 | 4 | 85.95 | 79.85 | 82.82 |  | 86.07 | 79.88 | 82.90 |  | 85.50 | 80.81 | 83.14 |  | 84.12 | 78.65 | 81.28 |
| 4 | 5 | 84.40 | 79.30 | 81.78 |  | 84.54 | 79.42 | 81.91 |  | 85.12 | 80.36 | 82.69 |  | 83.79 | 77.79 | 80.73 |
| 5 | 1 | 88.78 | 80.70 | 84.69 |  | 88.17 | 80.30 | 84.18 |  | 89.03 | 81.86 | 85.08 |  | 87.77 | 79.52 | 83.62 |
| 5 | 2 | 89.08 | 80.46 | 84.72 |  | 88.90 | 80.59 | 84.71 |  | 88.51 | 81.38 | 84.95 |  | 87.08 | 80.03 | 83.52 |
| 5 | 3 | 87.36 | 80.99 | 84.13 |  | 87.41 | 80.74 | 84.04 |  | 86.53 | 81.58 | 83.98 |  | 85.02 | 79.76 | 82.36 |
| 5 | 4 | 80.09 | 81.09 | 83.08 |  | 85.97 | 79.95 | 82.92 |  | 85.25 | 80.62 | 82.88 |  | 83.94 | 77.66 | 80.77 |
| 5 | 5 | 84.45 | 80.12 | 82.26 |  | 84.49 | 79.97 | 82.22 |  | 85.37 | 79.07 | 82.20 |  | 82.04 | 76.84 | 79.39 |
| 6 | 1 | 87.47 | 80.60 | 83.98 |  | 88.42 | 80.64 | 84.47 |  | 89.04 | 81.63 | 85.29 |  | 88.02 | 80.66 | 84.25 |
| 6 | 2 | 86.09 | 78.74 | 82.39 |  | 89.13 | 81.15 | 85.09 |  | 87.62 | 81.54 | 84.57 |  | 86.68 | 79.48 | 83.01 |
| 6 | 3 | 84.72 | 79.58 | 82.19 |  | 87.67 | 81.35 | 84.47 |  | 85.96 | 80.79 | 83.31 |  | 84.99 | 77.30 | 81.11 |
| 6 | 4 | 88.71 | 75.78 | 82.29 |  | 85.85 | 80.64 | 83.20 |  | 85.36 | 79.62 | 82.43 |  | 81.84 | 76.85 | 79.27 |
| 6 | 5 | 88.64 | 78.29 | 83.33 |  | 84.82 | 80.14 | 82.43 |  | 85.46 | 77.79 | 81.53 |  | 82.27 | 74.39 | 78.29 |
| 7 | 1 | 88.78 | 78.85 | 83.85 |  | 88.66 | 81.21 | 84.87 |  | **89.03** | **81.85** | **85.42** |  | **87.99** | **80.83** | **84.4** |
| 7 | 2 | 87.03 | 80.14 | 83.08 |  | 89.27 | 80.89 | 84.06 |  | 87.10 | 80.54 | 83.75 |  | 86.14 | 78.14 | 82.12 |
| 7 | 3 | 84.41 | 79.69 | 81.46 |  | 86.97 | 81.48 | 84.23 |  | 85.67 | 79.38 | 82.49 |  | 83.61 | 76.54 | 80.04 |
| 7 | 4 | 85.31 | 84.16 | 84.72 |  | 85.02 | 80.61 | 82.77 |  | 85.19 | 76.98 | 81.03 |  | 82.34 | 74.59 | 78.39 |
| 7 | 5 | 84.02 | 79.36 | 81.56 |  | 85.27 | 79.53 | 82.33 |  | 84.38 | 75.78 | 80.01 |  | 82.51 | 72.21 | 77.31 |
| **Average** | | **86.69** | **79.95** | **83.34** |  | **87.11** | **80.38** | **83.70** |  | **86.88** | **80.15** | **83.47** |  | **84.90** | **77.68** | **81.24** |

Table 2 The best *m* when λ=1 for each patient.

| **Patient ID** | **Parameters** | | **SPE** | | |  | **Patient ID** | **Parameters** | | **TPE** | | |
| --- | --- | --- | --- | --- | --- | --- | --- | --- | --- | --- | --- | --- |
|  | m | λ | Sensitivity | Specificity | Accuracy |  |  | m | λ | Sensitivity | Specificity | Accuracy |
| 1 | 4 | 1 | 84.72 | 79.58 | 82.19 |  | 1 | 7 | 1 | 84.72 | 80.56 | 82.60 |
| 2 | 6 | 1 | 85.53 | 92.26 | 88.75 |  | 2 | 7 | 1 | 86.28 | 92.35 | 89.17 |
| 3 | 3 | 1 | 71.09 | 87.21 | 79.17 |  | 3 | 3 | 1 | 70.39 | 87.38 | 78.92 |
| 4 | 3 | 1 | 99.84 | 98.54 | 99.17 |  | 4 | 3 | 1 | 99.84 | 98.54 | 99.17 |
| 5 | 4 | 1 | 91.84 | 92.08 | 91.92 |  | 5 | 3 | 1 | 91.33 | 92.24 | 91.75 |
| 6 | 4 | 1 | 98.02 | 99.15 | 98.61 |  | 6 | 3 | 1 | 98.02 | 99.43 | 98.75 |
| 7 | 3 | 1 | 100.00 | 98.94 | 99.44 |  | 7 | 7 | 1 | 100.00 | 99.19 | 99.58 |
| 9 | 4 | 1 | 69.59 | 64.93 | 67.33 |  | 9 | 5 | 1 | 73.14 | 62.90 | 67.83 |
| 10 | 4 | 1 | 99.00 | 76.28 | 87.67 |  | 10 | 3 | 1 | 99.02 | 77.17 | 88.08 |
| 11 | 5 | 1 | 93.38 | 55.79 | 74.48 |  | 11 | 7 | 1 | 93.51 | 55.59 | 74.48 |
| 12 | 3 | 1 | 100.00 | 99.60 | 99.79 |  | 12 | 4 | 1 | 100.00 | 100.00 | 100.00 |
| 14 | 4 | 1 | 84.29 | 75.75 | 80.00 |  | 14 | 4 | 1 | 83.91 | 78.92 | 81.35 |
| 15 | 3 | 1 | 91.62 | 92.21 | 91.98 |  | 15 | 7 | 1 | 91.63 | 93.18 | 92.40 |
| 16 | 5 | 1 | 87.43 | 76.12 | 81.75 |  | 16 | 7 | 1 | 87.13 | 78.46 | 82.67 |
| 17 | 4 | 1 | 92.98 | 65.44 | 79.25 |  | 17 | 3 | 1 | 92.98 | 65.11 | 79.08 |
| 18 | 3 | 1 | 94.21 | 55.29 | 74.67 |  | 18 | 3 | 1 | 94.21 | 54.78 | 74.42 |
| 19 | 3 | 1 | 88.78 | 78.85 | 83.85 |  | 19 | 3 | 1 | 88.73 | 79.05 | 83.96 |
| 20 | 5 | 1 | 97.22 | 95.31 | 96.33 |  | 20 | 7 | 1 | 97.77 | 95.13 | 96.50 |
| 21 | 4 | 1 | 86.29 | 64.49 | 75.33 |  | 21 | 3 | 1 | 86.29 | 64.49 | 75.33 |
|  |  |  |  |  |  |  |  |  |  |  |  |  |
| **Patient ID** | **Parameters** | | **RPE** | | |  | **Patient ID** | **Parameters** | | **MPE** | | |
|  | m | λ | Sensitivity | Specificity | Accuracy |  |  | m | λ | Sensitivity | Specificity | Accuracy |
| 1 | 5 | 1 | 86.01 | 77.64 | 81.77 |  | 1 | 6 | 1 | 82.21 | 77.70 | 79.58 |
| 2 | 7 | 1 | 85.45 | 90.64 | 88.33 |  | 2 | 5 | 1 | 83.33 | 92.70 | 87.92 |
| 3 | 5 | 1 | 74.40 | 86.28 | 80.25 |  | 3 | 5 | 1 | 71.91 | 84.77 | 78.42 |
| 4 | 4 | 1 | 99.84 | 98.49 | 99.17 |  | 4 | 6 | 1 | 99.85 | 98.64 | 99.25 |
| 5 | 7 | 1 | 93.71 | 90.21 | 91.92 |  | 5 | 7 | 1 | 94.04 | 90.59 | 92.25 |
| 6 | 7 | 1 | 98.10 | 100.00 | 99.03 |  | 6 | 6 | 1 | 98.10 | 100.00 | 99.03 |
| 7 | 3 | 1 | 100.00 | 97.89 | 98.89 |  | 7 | 4 | 1 | 99.15 | 91.55 | 95.28 |
| 9 | 7 | 1 | 67.19 | 70.77 | 68.75 |  | 9 | 7 | 1 | 65.52 | 70.23 | 67.83 |
| 10 | 5 | 1 | 99.31 | 72.41 | 85.92 |  | 10 | 6 | 1 | 99.32 | 71.43 | 85.42 |
| 11 | 6 | 1 | 93.27 | 62.77 | 78.02 |  | 11 | 7 | 1 | 92.14 | 60.98 | 76.67 |
| 12 | 3 | 1 | 100.00 | 99.60 | 99.79 |  | 12 | 5 | 1 | 100.00 | 99.59 | 99.79 |
| 14 | 5 | 1 | 83.06 | 75.51 | 79.38 |  | 14 | 7 | 1 | 77.66 | 73.02 | 75.31 |
| 15 | 7 | 1 | 94.00 | 94.37 | 94.17 |  | 15 | 7 | 1 | 93.34 | 92.77 | 92.92 |
| 16 | 7 | 1 | 88.10 | 80.75 | 84.42 |  | 16 | 7 | 1 | 88.68 | 79.39 | 84.00 |
| 17 | 7 | 1 | 89.47 | 74.46 | 81.92 |  | 17 | 7 | 1 | 89.17 | 73.60 | 81.42 |
| 18 | 7 | 1 | 91.48 | 63.61 | 77.33 |  | 18 | 7 | 1 | 88.76 | 66.90 | 77.83 |
| 19 | 3 | 1 | 89.15 | 77.78 | 83.33 |  | 19 | 7 | 1 | 87.80 | 71.94 | 79.79 |
| 20 | 7 | 1 | 98.03 | 96.21 | 97.08 |  | 20 | 7 | 1 | 97.36 | 95.50 | 96.42 |
| 21 | 7 | 1 | 86.55 | 73.27 | 79.83 |  | 21 | 7 | 1 | 87.17 | 72.73 | 79.92 |

Table 3 The number of patients when they got the best results by using specific *m*.

| **Parameters** | | **SPE** | **TPE** | **RPE** | **MPE** |
| --- | --- | --- | --- | --- | --- |
| **m** | **λ** |
| 3 | 1 | **7** | **9** | 3 | 0 |
| 4 | 1 | **8** | 2 | 1 | 1 |
| 5 | 1 | 3 | 1 | 4 | 3 |
| 6 | 1 | 1 | 0 | 1 | 4 |
| 7 | 1 | 0 | **7** | **10** | **11** |

Table 4 The average results for all patients when using different q.

| **TPE（m=3,λ=1）** | | | | **RPE（m=7,λ=1）** | | | |
| --- | --- | --- | --- | --- | --- | --- | --- |
| **q** | **Sensitivity** | **Specificity** | **Accuracy** | **q** | **Sensitivity** | **Specificity** | **Accuracy** |
| 0.1 | 83.93 | 77.77 | 80.80 | 0.1 | 83.67 | 77.62 | 80.56 |
| 0.2 | 84.41 | 78.73 | 81.52 | 0.2 | 83.94 | 78.50 | 81.17 |
| 0.3 | 85.17 | 79.42 | 82.21 | 0.3 | 84.72 | 79.32 | 81.96 |
| 0.4 | 85.88 | 79.89 | 82.85 | 0.4 | 85.48 | 79.85 | 82.59 |
| 0.5 | 86.53 | 80.20 | 83.36 | 0.5 | 85.88 | 80.45 | 83.09 |
| 0.6 | 87.51 | 80.20 | 83.81 | 0.6 | 86.51 | 80.50 | 83.47 |
| 0.7 | 88.06 | 80.43 | 84.22 | 0.7 | 87.12 | 80.72 | 83.89 |
| 0.8 | 88.36 | 80.88 | 84.60 | 0.8 | 87.45 | 81.48 | 84.46 |
| 0.9 | 88.77 | 80.93 | 84.80 | 0.9 | 87.93 | 81.60 | 84.70 |
| **2** | **89.24** | **81.73** | **85.45** | **2** | **88.66** | **82.02** | **85.31** |
| 3 | 88.42 | 80.93 | 84.66 | 3 | 88.37 | 81.82 | 85.04 |
| 4 | 88.29 | 79.31 | 83.76 | 4 | 88.48 | 81.76 | 85.09 |
| 5 | 86.27 | 78.06 | 82.09 | 5 | 88.54 | 81.65 | 85.08 |
| 6 | 83.95 | 76.29 | 80.05 | 6 | 88.32 | 81.72 | 84.97 |
| 7 | 81.28 | 74.68 | 77.82 | 7 | 88.15 | 81.53 | 84.84 |
| 8 | 79.59 | 72.55 | 75.78 | 8 | 88.20 | 81.55 | 84.82 |
| 9 | 78.36 | 70.09 | 73.94 | 9 | 88.13 | 81.36 | 84.71 |

Table 5 The prediction results for all patients by using different parameter pairs for SPE.

| **Patient ID** | **SPE（m=4,λ=1）** | | | | **SPE（m=3,λ=1）** | | | |
| --- | --- | --- | --- | --- | --- | --- | --- | --- |
|  | **SS** | **FPR** | **Average SPH (min)** | **Distance** | **SS** | **FPR** | **Average SPH (min)** | **Distance** |
| 1 | 1.000 | 0.281 | 59.00 | 15.25 | 0.94 | 0.16 | 57.78 | 10.53 |
| 2 | 0.708 | 0.042 | 49.90 | 29.28 | 1.00 | 0.29 | 48.90 | 10.94 |
| 3 | 1.000 | 0.125 | 63.02 | 9.26 | 1.00 | 0.13 | 61.22 | 9.26 |
| 4 | 1.000 | 0.000 | 60.17 | 0.00 | 1.00 | 0.00 | 60.17 | 0.00 |
| 5 | 0.800 | 0.525 | 55.75 | 36.42 | 0.73 | 0.63 | 51.04 | 38.87 |
| 6 | 1.000 | 0.167 | 59.76 | 6.67 | 0.75 | 0.46 | 60.34 | 31.79 |
| 7 | 1.000 | 0.042 | 63.26 | 4.17 | 1.00 | 0.00 | 64.51 | 0.00 |
| 9 | 1.000 | 0.000 | 67.21 | 0.00 | 0.90 | 0.18 | 49.24 | 15.67 |
| 10 | 1.000 | 0.000 | 75.57 | 0.00 | 0.83 | 0.00 | 79.14 | 17.50 |
| 11 | 0.781 | 0.063 | 54.23 | 22.04 | 0.75 | 0.06 | 57.11 | 25.17 |
| 12 | 1.000 | 0.125 | 34.50 | 26.67 | 1.00 | 0.13 | 53.72 | 9.26 |
| 14 | 1.000 | 0.063 | 48.87 | 4.65 | 0.97 | 0.29 | 61.42 | 3.13 |
| 15 | 1.000 | 0.000 | 59.38 | 0.00 | 1.00 | 0.00 | 59.94 | 0.00 |
| 16 | 0.800 | 0.200 | 58.63 | 24.89 | 0.95 | 0.38 | 46.01 | 22.94 |
| 17 | 1.000 | 0.000 | 77.66 | 0.00 | 1.00 | 0.00 | 81.26 | 0.00 |
| 18 | 0.925 | 0.075 | 64.29 | 10.89 | 1.00 | 0.00 | 68.88 | 0.00 |
| 19 | 0.875 | 0.250 | 65.55 | 17.85 | 0.97 | 0.28 | 66.94 | 12.40 |
| 20 | 0.975 | 0.150 | 79.35 | 9.70 | 1.00 | 0.23 | 79.39 | 11.54 |
| 21 | 1.000 | 0.000 | 80.53 | 0.00 | 0.80 | 0.38 | 68.82 | 27.75 |
| **Average** | **0.940** | **0.111** | **61.93** | **11.49** | **0.93** | **0.19** | **61.89** | **12.99** |

Table 6 The prediction results for all patients for TPE, RPE and MPE.

| **Patient ID** | **TPE（m=3,λ=1,q=2）** | | | | **RPE（m=7,λ=1,q=2）** | | | | | **MPE（m=7,λ=1）** | | | |
| --- | --- | --- | --- | --- | --- | --- | --- | --- | --- | --- | --- | --- | --- |
|  | **SS** | **FPR** | **Average SPH (min)** | **Distance** | **SS** | **FPR** | **Average SPH (min)** | | **Distance** | **SS** | **FPR** | **Average SPH (min)** | **Distance** |
| 1 | 0.97 | 0.16 | 48.35 | 8.77 | 0.97 | 0.09 | 48.93 | 6.04 | | 0.97 | 0.16 | 54.37 | 8.19 |
| 2 | 0.92 | 0.21 | 46.86 | 11.69 | 0.92 | 0.17 | 45.50 | 11.15 | | 1.00 | 0.17 | 45.15 | 6.45 |
| 3 | 0.98 | 0.10 | 51.91 | 7.95 | 1.00 | 0.15 | 61.37 | 13.33 | | 1.00 | 0.13 | 60.32 | 14.29 |
| 4 | 1.00 | 0.00 | 60.17 | 0.00 | 1.00 | 0.00 | 60.17 | 0.00 | | 1.00 | 0.00 | 60.17 | 0.00 |
| 5 | 0.63 | 0.53 | 39.17 | 44.91 | 0.68 | 0.48 | 45.06 | 40.81 | | 0.65 | 0.55 | 42.71 | 44.95 |
| 6 | 0.79 | 0.58 | 58.48 | 32.54 | 0.67 | 0.00 | 64.75 | 33.33 | | 0.71 | 0.38 | 63.83 | 33.17 |
| 7 | 1.00 | 0.00 | 63.51 | 0.00 | 1.00 | 0.00 | 62.01 | 0.00 | | 1.00 | 0.00 | 63.26 | 0.00 |
| 9 | 0.93 | 0.10 | 45.48 | 10.27 | 0.95 | 0.05 | 47.84 | 5.85 | | 0.90 | 0.28 | 52.11 | 18.44 |
| 10 | 0.83 | 0.03 | 78.60 | 17.63 | 0.80 | 0.00 | 80.72 | 20.00 | | 0.80 | 0.00 | 76.22 | 20.00 |
| 11 | 0.75 | 0.06 | 56.86 | 25.16 | 0.88 | 0.31 | 57.98 | 16.72 | | 0.81 | 0.19 | 56.42 | 20.10 |
| 12 | 0.96 | 0.18 | 49.24 | 15.67 | 1.00 | 0.17 | 75.51 | 10.00 | | 0.50 | 0.00 | 64.60 | 50.00 |
| 14 | 0.94 | 0.33 | 49.99 | 6.25 | 1.00 | 0.33 | 63.49 | 5.85 | | 0.91 | 0.48 | 63.14 | 9.38 |
| 15 | 1.00 | 0.00 | 56.38 | 0.00 | 1.00 | 0.00 | 55.82 | 0.00 | | 1.00 | 0.00 | 52.25 | 0.00 |
| 16 | 0.93 | 0.43 | 50.69 | 26.83 | 0.95 | 0.20 | 47.84 | 12.63 | | 1.00 | 0.23 | 44.48 | 12.16 |
| 17 | 1.00 | 0.00 | 80.96 | 0.00 | 1.00 | 0.00 | 83.81 | 0.00 | | 1.00 | 0.00 | 89.96 | 0.00 |
| 18 | 1.00 | 0.00 | 68.28 | 0.00 | 1.00 | 0.00 | 71.28 | 0.00 | | 1.00 | 0.00 | 61.44 | 0.00 |
| 19 | 0.97 | 0.22 | 62.49 | 9.19 | 0.94 | 0.22 | 64.96 | 11.67 | | 0.78 | 0.19 | 73.27 | 23.26 |
| 20 | 0.98 | 0.23 | 80.42 | 11.52 | 0.98 | 0.18 | 81.04 | 8.43 | | 0.95 | 0.13 | 82.94 | 7.43 |
| 21 | 0.98 | 0.34 | 82.19 | 2.50 | 0.93 | 0.48 | 73.52 | 23.33 | | 0.90 | 0.50 | 66.24 | 26.93 |
| **Average** | **0.92** | **0.17** | **59.47** | **12.15** | **0.93** | **0.15** | **62.72** | **11.53** | | **0.89** | **0.18** | **61.73** | **15.51** |
